# Cell-Free DNA at Diagnosis for Stage IV Non-Small Cell Lung Cancer: Costs, Time to Diagnosis and Clinical Relevance

**DOI:** 10.3390/cancers14071783

**Published:** 2022-03-31

**Authors:** Simone N. Koole, Daan C. L. Vessies, Milou M. F. Schuurbiers, Astrid Kramer, Robert D. Schouten, Koen Degeling, Linda J. W. Bosch, Michel M. van den Heuvel, Wim H. van Harten, Daan van den Broek, Kim Monkhorst, Valesca P. Retèl

**Affiliations:** 1 Division of Psychosocial Research and Epidemiology, The Netherlands Cancer Institute, 1066 CX Amsterdam, The Netherlands; s.koole@nki.nl (S.N.K.); w.v.harten@nki.nl (W.H.v.H.); 2 Department of Laboratory Medicine, Netherlands Cancer Institute, 1066 CX Amsterdam, The Netherlands; d.vessies@nki.nl; 3 Department of Pulmonary Diseases, Radboud University Medical Centre, 6525 XZ Nijmegen, The Netherlands; milou.schuurbiers@radboudumc.nl (M.M.F.S.); michel.vandenheuvel@radboudumc.nl (M.M.v.d.H.); 4 Department of Epidemiology and Data Science, Amsterdam University Medical Centers (Location VUmc), 1081 HV Amsterdam, The Netherlands; a.kramer@nki.nl; 5 Department of Pulmonology, The Netherlands Cancer Institute, 1066 CX Amsterdam, The Netherlands; rd.schouten@nki.nl; 6 Cancer Health Services Research, Centre for Health Policy & Centre for Cancer Research, Faculty of Medicine, Dentistry and Health Sciences, The University of Melbourne, Melbourne VIC 3010, Australia; koen.degeling@unimelb.edu.au; 7 Department of Pathology, Netherlands Cancer Institute, 1066 CX Amsterdam, The Netherlands; l.bosch@nki.nl (L.J.W.B.); k.monkhorst@nki.nl (K.M.); 8 Department of Health Technology and Services Research University of Twente, 7522 NB Enschede, The Netherlands; 9 Department of Clinical Chemistry, The Netherlands Cancer Institute, 1066 CX Amsterdam, The Netherlands; da.vd.broek@nki.nl

**Keywords:** non-small-cell lung cancer, cfDNA, molecular diagnostics, discrete event simulation

## Abstract

**Simple Summary:**

To obtain non-small-cell lung cancer (NSCLC) tissue, patients undergo burdensome procedures, such as needle biopsies or endoscopic or surgical procedures. Recent technological developments have enabled targeted sequencing using plasma. Next-generation sequencing (NGS) based on cfDNA can provide broad genetic information and comprehensive identification of relevant targets for therapy in a single test. Consecutive or combined use of liquid biopsy-based NGS can potentially result in more efficient and less invasive molecular profiling in NSCLC patients. Using a process-based discrete event simulation with data on stage IV NSCLC patients from the LEMA trial, this study aimed to estimate the effects on costs, throughput time, and diagnostic yield of two diagnostic scenarios with liquid biopsies, compared to diagnostics with tissue biopsies alone.

**Abstract:**

Tissue biopsies can be burdensome and are only effective in 10–30% of patients with metastasized non-small-cell lung cancer (mNSCLC). Next-generation sequencing (NGS) on cell-free DNA (cfDNA) might be an attractive alternative. We evaluated the costs, throughput time, and diagnostic yield of two diagnostic scenarios with tissue and cfDNA for mNSCLC patients, compared to diagnostics based on tissue biopsy alone. Data were retrieved from 209 stage IV NSCLC patients included in 10 hospitals in the Netherlands in the observational Lung cancer Early Molecular Assessment (LEMA) trial. Discrete event simulation was developed to compare three scenarios, using LEMA data as input where possible: (1) diagnostics with “tissue only”; (2) diagnostics with “cfDNA first”, and subsequent tissue biopsy if required (negative for EGFR, BRAF ALK, ROS1); (3) cfDNA if tissue biopsy failed (“tissue first”). Scenario- and probabilistic analyses were performed to quantify uncertainty. In scenario 1, 84% (Credibility Interval [CrI] 70–94%) of the cases had a clinically relevant test result, compared to 93% (CrI 86–98%) in scenario 2, and 93% (CrI 86–99%) in scenario 3. The mean throughput time was 20 days (CrI 17–23) pp in scenario 1, 9 days (CrI 7–11) in scenario 2, and 19 days (CrI 16–22) in scenario 3. Mean costs were €2304 pp (CrI €2067–2507) in scenario 1, compared to €3218 (CrI €3071–3396) for scenario 2, and €2448 (CrI €2382–2506) for scenario 3. Scenarios 2 and 3 led to a reduction in tissue biopsies of 16% and 9%, respectively. In this process-based simulation analysis, the implementation of cfDNA for patients with mNSCLC resulted in faster completion of molecular profiling with more identified targets, with marginal extra costs in scenario 3.

## 1. Introduction

Treatment of patients with metastatic non-small-cell lung cancer (mNSCLC) has evolved substantially over the past decade. Since the introduction of immunotherapy and targeted therapy, molecular diagnostics has become of great importance for these patients [1]. Standard diagnostics include tumor biopsies for pathology review and immunohistochemistry, combined with next-generation sequencing (NGS), FISH, and PCR tests [2,3,4]. To obtain NSCLC tissue, patients undergo burdensome procedures, such as needle biopsies or endoscopic or surgical procedures. Due to tumor localization, lung cancer biopsies have a relatively high failure rate of 10–30%, and up to half of the patients need multiple biopsies [5,6]. Significant complications like a pneumothorax are recorded in 10–25% of patients [5,7].

Peripheral blood sampling may be an alternative for tissue as a source of tumor material. It is minimally invasive, and cell-free DNA (cfDNA) analysis from plasma, in other words liquid biopsy analysis, can be used for cancer genomics [8]. In the case of cancer, a proportion of the tumor DNA is released into the blood of cancer patients after degradation and apoptosis of cancer cells. Recent technological developments enabled targeted sequencing using plasma, including the detection of point mutations, tyrosine kinase fusions, and copy number variations (CNVs). NGS based on cfDNA can provide broad genetic information and comprehensive identification of relevant targets for therapy in a single test [9,10].

The Lung cancer Early Molecular Assessment (LEMA) trial included 878 patients with NSCLC and performed upfront decentralized tissue molecular profiling [11]. For a subcohort of patients with confirmed stage IV mNSCLC, centralized cfDNA analyses was performed. In this cohort, the sensitivity of plasma cfDNA analysis was 68–80%, with a concordance of 87–98% in identified biomarkers compared to tissue molecular profiling [10,11,12].

Consecutive or combined use of liquid biopsy-based NGS can potentially result in a more efficient and less invasive process of molecular profiling in NSCLC patients. Using a process-based discrete event simulation using data from stage IV NSCLC patients from the LEMA trial, this study aimed to estimate the effects on costs, throughput time, and diagnostic yield of two diagnostic scenarios with liquid biopsies, compared to diagnostics with tissue biopsies alone. 

## 2. Materials and Methods

### 2.1. Clinical Data Retrieval

Clinical data were retrieved from 209 stage IV NSCLC patients who were included in the observational Lung cancer Early Molecular Assessment trial (LEMA; NCT02894853). Within this prospective, multicenter study, standard of care (SOC) tissue diagnostics were performed in 10 hospitals in the Netherlands. Molecular profiling was performed upfront, irrespective of disease stage and pathology, using both tissue and blood-based DNA testing. Plasma samples were collected at diagnosis, in parallel with tissue biopsies.

The current study used a subgroup of the LEMA cohort, consisting of the first consecutive cohort of 209 patients with confirmed stage IV non-small-cell lung cancer (NSCLC) for whom pretreatment plasma was available. This subcohort is the POPSTAR cohort. For the POPSTAR cohort, sequencing of cfDNA was performed centrally and retrospectively, using AVENIO cfDNA analysis kits (Roche Diagnostics, Pleasanton, CA, USA), and compared to results from SOC tissue diagnostics [11]. The primary outcome of the LEMA study was the percentage of patients with EGFR mutations or ALK translocations using both tumor tissue and cfDNA. Evaluation of the agreement between SOC and cfDNA was the secondary outcome of the trial. 

Because LEMA was an observational trial, and because of the retrospective batch-wise nature of the cfDNA analysis in the subcohort, the diagnostic trajectory with cfDNA, as it might be used for clinical decision making, could not be extracted from the data. Therefore, we designed two alternative, realistic scenarios for the implementation of cfDNA for diagnostics in patients with advanced stage lung cancer. 

### 2.2. Discrete Event Simulation

The aim of this simulation analysis was to study the effects of the implementation of cfDNA analysis in the diagnostic workup for patients with stage IV NSCLC. The outcomes of the analysis were: (1) proportion of patients with complete molecular profiling and failure rates, (2) throughput time from tissue/blood retrieval to final diagnosis, and (3) costs of the diagnostic workflow. Because of the retrospective nature of the cfDNA assessment in this observational patient cohort, the diagnostic trajectory with cfDNA analysis was modeled in three realistic scenarios, specified in Box 1.

Box 1 **Overview of the drafted scenarios.**

Based on expert elicitation three realistic scenarios were drafted:

(1) Diagnostic procedure with tissue biopsy alone (standard of care). Patients undergo 1, or 2 tissue biopsy attempts; in case of failure (“tissue only”);
(2) Diagnostic procedure starting with cfDNA, if no definitive result is retrieved, a tissue biopsy is still required; (“cfDNA first”); and
(3) Diagnostic procedure starting with tissue biopsy, if the first attempt failed, blood is collected for cfDNA analysis. performed (“tissue first”).

### 2.3. Input for the Model

#### 2.3.1. Patient Data

From the LEMA dataset, clinical data from 209 confirmed stage IV patients were used in this analysis. Data on clinical stage and diagnostic resources used in SOC diagnostics (e.g., immunohistochemistry, FISH, SISH, DISH, rtPCR, and NGS) were collected for each patient. Success rates, molecular analysis results, and identified targets were collected from the patient electronic case report forms from the LEMA trial, for both tissue and cfDNA analysis. Because information was lacking on the number of attempts at tissue retrieval, the percentage of patients who needed two biopsy attempts was based on a selection of the LEMA patients within the Netherlands Cancer Institute. Test results for both tissue and cfDNA were evaluated per patient by an expert team (RS, DV, LB, KM, and MvdH) in order to evaluate the clinical relevance (implications for registered treatment) and consequences in terms of current potential targeted treatment options [11]. Molecular diagnostics in the model focused on the detection of nine specific oncogenes (EGFR, BRAF, ALK, ROS, KRAS, MET, RET, NRAS, and ERBB2) and PDL1 immunohistochemistry. 

#### 2.3.2. Throughput Time

The throughput time for tissue analysis was estimated based on historic data and expert elicitations (LB, KM). We made several assumptions in the estimation of throughput times:

(1) Immunohistochemical (IHC) staining was performed in parallel with NGS. The mean number of days between tissue arrival and request for IHC and NGS was three days. The throughput time of NGS was estimated based on the mean throughput time of eight days, measured by the pathology department of the Netherlands Cancer Institute for isolation and analysis.
(2) Complementary molecular diagnostics (e.g., rtPCR/FISH) after NGS were performed in parallel. The mean throughput time of rtPCR and FISH combined was estimated to be five days, based on estimations of the pathology department of the Netherlands Cancer Institute.
(3) The throughput time for cfDNA sequencing including blood withdrawal was estimated based on expert opinions (LB, DV), and set to a mean of 7.5 days. The assumption was made that isolation and sequencing for cfDNA analysis would be performed once a week.

#### 2.3.3. Costs of Tissue-Based Diagnostics

Costs were based on a published Dutch microcosting analysis [13]. Costs for machinery, material, personnel, and small equipment for each diagnostic test in standard diagnostics for lung cancer were incorporated in the cost analysis. Sample registration, preparation, fixation, embedding, cutting, isolation, and evaluation were measured by the Netherlands Cancer Institute, and were included. RNA-NGS for ALK, ROS1, RET, NTRK, and METex14 was not included. Personnel costs included costs for all staff (i.e., technicians, pathologists, and clinical molecular pathologists). The cost of the platforms, software, and consumables all included 21% value-added tax (VAT). Costs for all individual tests were listed separately. For the costs for overhead and housing, a markup of 44% was charged [14]. We assumed that these covered the costs for multipurpose equipment and all other indirect costs. The costs for individual tests were linked to the steps in the model. Process-based cost calculations for diagnostic applications can be found in supplementary Table S1.

All costs for tissue retrieval, and preparation are based on the hospital billing costs of a single institute (Netherlands Cancer Institute). Costs for tissue retrieval and biopsies were based on the national tariff for endobronchial ultrasound or a computed tomography-guided biopsy [14,15].

#### 2.3.4. Costs of cfDNA Analysis

Costs for cfDNA analysis were based on a recent microcosting analysis.(unpublished) The microcosting analysis was an activity-based costing method, and includes the costs for personnel, materials, and equipment. Personnel costs include costs for all staff (i.e., technicians, pathologists, and clinical molecular pathologists). Several assumptions were made to calculate the cost price. The cost price was based on analyzing 12 samples per week in one run, assuming that not all runs will be completely full. In line with the costing of tissue-based diagnostics, a 44% markup was used to account for overhead [14]. We assumed that these covered the costs for multipurpose equipment and all other indirect costs. A 0.5% failure rate was included in the costs [11]. Utilization of equipment was assumed to be 33%. Steps of the microcosting framework include sample collection, internal sample transport, sample processing, cfDNA isolation with QiaSymphony SP (Qiagen, Germantown, MD, USA), ctDNA analysis with Avenio ctDNA Targeted Kit (Roche Diagnostics, Pleasanton, CA, USA) + NextSeq 550 (Illumina, San Diego, CA, USA), and reporting of results. Costs are listed in supplementary Table S2. Costs for material and equipment include 21% VAT. 

### 2.4. Definitions of Success and Output of the Model

- A “clinically relevant test result” is defined as a simulated patient who has a negative or positive test result after molecular diagnostics without any failures. This includes patients with a “complete test result,” but also patients with a nearly “complete result.” For example, a cfDNA test result positive for KRAS but without biopsy PDL1 staining is considered clinically relevant. See the definitions of the possible diagnostic results below:
  - “Complete Test Result”: Patients have successfully undergone all required tests for the previously mentioned oncogenes and PDL1, and there is a complete result. This also applies to patients with all required tests performed, but negative results for all 9 oncogenes and PDL1 staining.
    The proportion of patients with “a positive test result for a biomarker” means the simulated cases for which a biomarker is found and indicates the proportion of simulated patients with an oncogenic EGFR, BRAF, ALK, ROS, KRAS, METe14, RET, NRAS, or ERBB2 mutation (including all oncogenic BRAF and KRAS variants).
  - ”Incomplete test result: cfDNA targets found but no PDL1 status (failed biopsy)” applies to patients with a complete molecular profile, or a result based on cfDNA but incomplete subsequent tissue analysis (e.g., cfDNA positive for KRAS but no PDL1 staining available). This is considered clinically relevant
- If there is no test result for the nine oncogenes (EGFR, BRAF, ALK, ROS, KRAS, METe14, RET, NRAS, and ERBB2) due to failure of biopsies or failure of NGS, then a simulated patient is considered to have “no conclusive molecular result.”

### 2.5. Model Description

#### 2.5.1. Input Data

Data were collected from 209 stage IV NSCLC patients from the LEMA cohort (11). All input parameters are listed in Supplementary Table S3. A few assumptions were made. It was assumed that in the “biopsy/tissue first” group, patients would not undergo a second biopsy, because of the alternative option for cfDNA analysis. Additionally, if an EGFR, BRAF, ALK, or ROS1 aberration was found with cfDNA, it was assumed that pulmonologists would be confident to start targeted therapy without histological or cytological confirmation. Furthermore, it was assumed that cfDNA analysis cannot identify all predictive biomarkers since some are analyzed with immunohistochemistry (e.g., PD-L1 expression) and/or with mismatch repair (MMR) analysis on tissue. As a consequence of these final two assumptions, patients with a KRAS, ERBB2, RET, or METe14 mutation can still undergo a tissue biopsy after cfDNA analysis (scenario 2).

#### 2.5.2. Discrete Event Simulation (DES)

In the DES, individual patients underwent a series of processes (events) affecting the outcome and costs of the diagnostic trajectory. The steps and subsequent events in each scenario were considered by an expert team (KM, MS, RS, DV, and SK). The probabilities of events happening, the cost of a step, and the duration of a step were based on observed data from the subcohort of stage IV patients of the LEMA trial or on the assumptions and expert opinions indicated above [11]. Probabilities of test outcomes were based directly on data from the POPSTAR cohort. The simulation and analyses described below were implemented based on previously described methods by Degeling et al. and performed using R statistics, Version 1.1.456 [16,17]. The simulation was internally validated to ensure that the probabilities of events happening in the simulation matched those observed in the data. 

**Figure 1:**
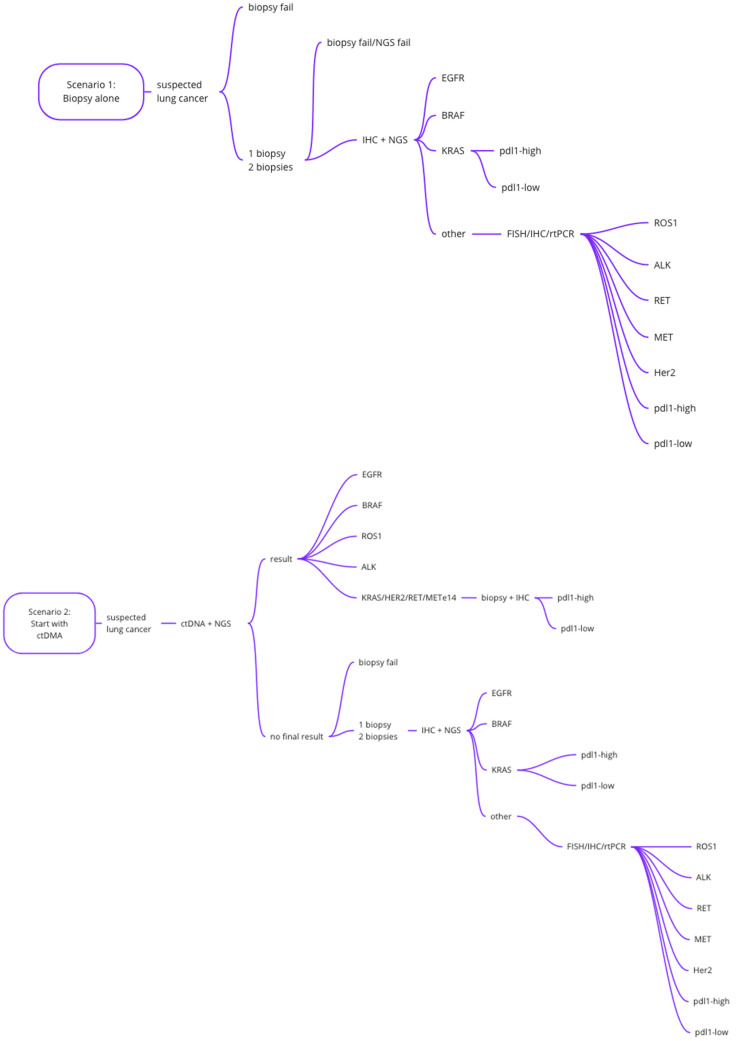

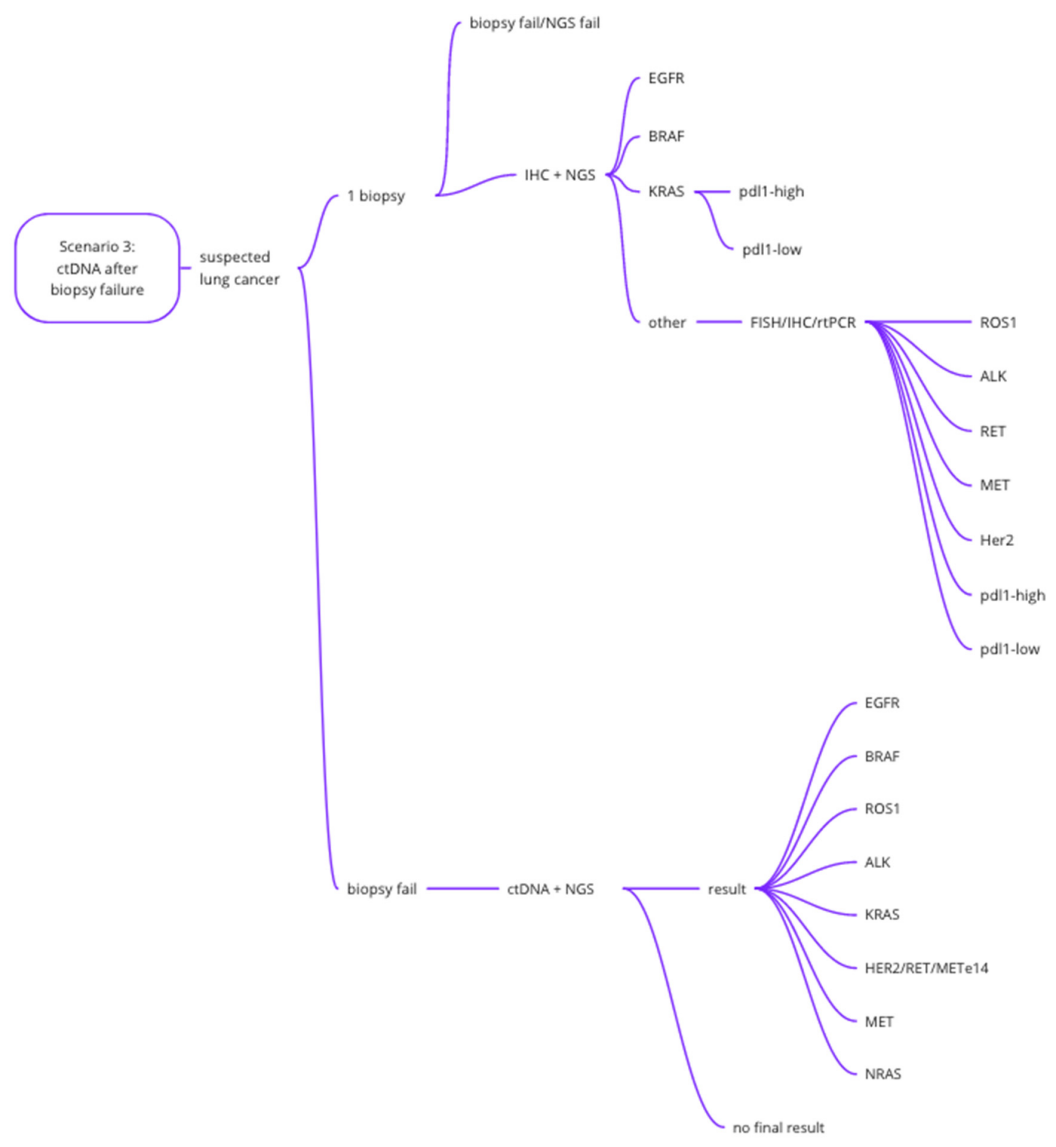
Schematic overview of the scenarios that are modeled in the discrete event simulation model.

A schematic overview of the three scenarios is plotted in Figure 1. In scenario 1, the success rate of the first biopsy is 77%. For 40% of patients with a failed first biopsy, a second biopsy is attempted, resulting in an overall biopsy success rate of 85%. 

#### 2.5.3. Deterministic Analysis

The means of all input parameters were used as input for the base-case model (Supplementary Table S3). Given that the costs and time parameters could not be measured directly from the study, and therefore were based on the literature and assumptions, a standard deviation of 17% for all cost and time variables was assumed based on expert input (SK, LB, and KM) to take into account stochastic uncertainty, i.e., the variation between patients that is not explained by heterogeneity, using Gamma distributions defined by the method of moments (Supplementary Table S4). In each run of the model, 10,000 patients were simulated per scenario to obtain stable outcome estimates (Supplementary Figure S1). This deterministic analysis provided insights into the uncertainty in the patient-level outcome.

#### 2.5.4. Probabilistic Analysis

A probabilistic analysis was performed to quantify the uncertainty in the mean outcomes of all patients. Parametric distributions were specified to define the uncertainty in relevant model parameters (Supplementary Table S3, Supplementary Figures S2 and S7) [18] Gamma distributions were defined for cost-and time-related parameters using the method of moments based on a standard error of 10%. Beta distributions were used for binary probabilities, such as the failure and/or success of biopsies (Supplementary Table S3). Dirichlet distributions were used for probabilities of more than two categories, such as the probabilities of test outcomes (e.g., probability of finding targets per test, Supplementary Figures S2). To perform a probabilistic analysis with 500 runs, with 10,000 simulated patients for each scenario in each run, we used a different set of sampled parameter values for each run. This number of runs was sufficient to obtain stable outcomes (Supplementary Figures S6 and S7).

#### 2.5.5. Scenario Analysis

A scenario analysis was performed to assess the impact of the overall biopsy failure rate on the outcomes. Whereas the base-case value for the overall biopsy failure rate was 15%, including patients who might have had two biopsy attempts, the scenario analysis considered alternative values of 1% and 30%. For each biopsy failure rate scenario, a full probabilistic analysis was performed of 500 runs with 10,000 simulated patients per strategy per run.

## 3. Results

### 3.1. Deterministic Analysis

The base-case results of the model are presented in Table 1. Eighty-four percent of the cases in scenario 1 had a clinically relevant test result, compared to 92% when cfDNA was added in scenario 2 (cfDNA first), and 88% in scenario 3 (cfDNA if tissue failed). Median throughput time was 20 days (interquartile range [IQR 17–23]) pp in scenario 1, and 10 days (IQR 7–25) and 18 (IQR 16–22) days in scenario 3. Mean costs were €2294 pp (standard deviation (SD): €868) in scenario 1, compared to €3350 (SD €1257) for scenario 2 and €2443 (SD €592) for scenario 3.

### 3.2. Probabilistic Analysis

**Figure 2:**
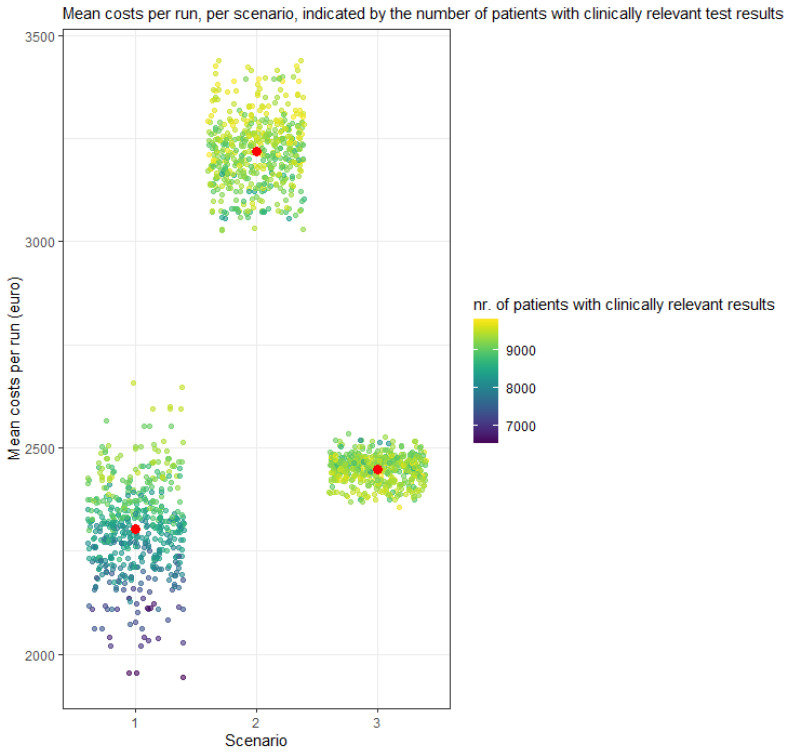
PSA outcomes: Means costs per run. One dot represents the mean costs of one run with 10,000 cases. The higher the number of cases with a clinically relevant result, the lighter the dot. Red = Mean total costs of all runs.

**Figure 3:**
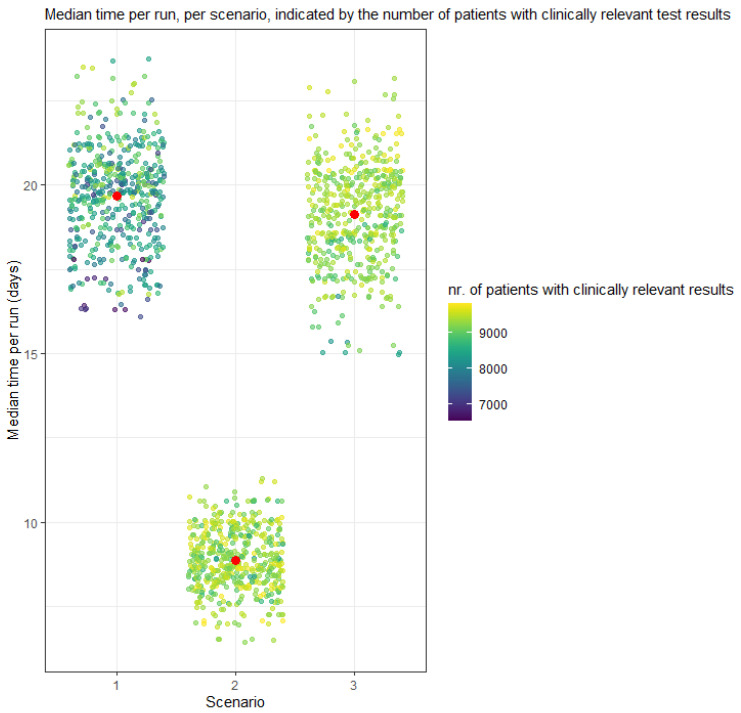
PSA outcomes: Means time per run. One dot represents the median time of one run with 10,000 cases. The higher the number of cases with a clinically relevant result, the lighter the dot. Red = Mean time of all runs.

Mean costs were €2304 pp (95% credibility interval (CrI): €2067–2507) in the “tissue-only” scenario, compared to €3218 (CrI €3071–3396) for the “cfDNA first” scenario, and €2448 pp (CrI €2382–2506) for “tissue biopsy first” scenario (Table 2). The scatterplot in Figure 2 presents the mean costs for all 500 runs per scenario, associated with the number of patients with clinically relevant test results per run. The median throughput time was 20 days (CrI 17–23) pp in scenario 1, 9 days (CrI 7–11) in scenario 2, and 19 (IQR 16–22) days in scenario 3. Figure 3 displays the throughput time for all runs, per scenario and per relevant test result. In the ‘tissue-only’ scenario 1, 84% (CrI 70–94%) of the cases had a clinically relevant test result, compared to 93% (CrI 86–98%) in scenario 2 and 93% (CrI 86–99%) in scenario 3. 

Regarding the mean number of biopsy attempts per scenario, scenario 1 resulted in an average of 1.10 biopsy attempts pp (CrI 0.67–1.28), scenario 2 in 0.92 attempts pp (CrI 0.70–1.03), and scenario 3 in 1.00 attempts pp. Hence, scenarios 2 and 3 led to a relative reduction in biopsies of 16% and 9%, respectively. (Supplementary Table S5)

### 3.3. Scenario Analysis

**Figure 4:**
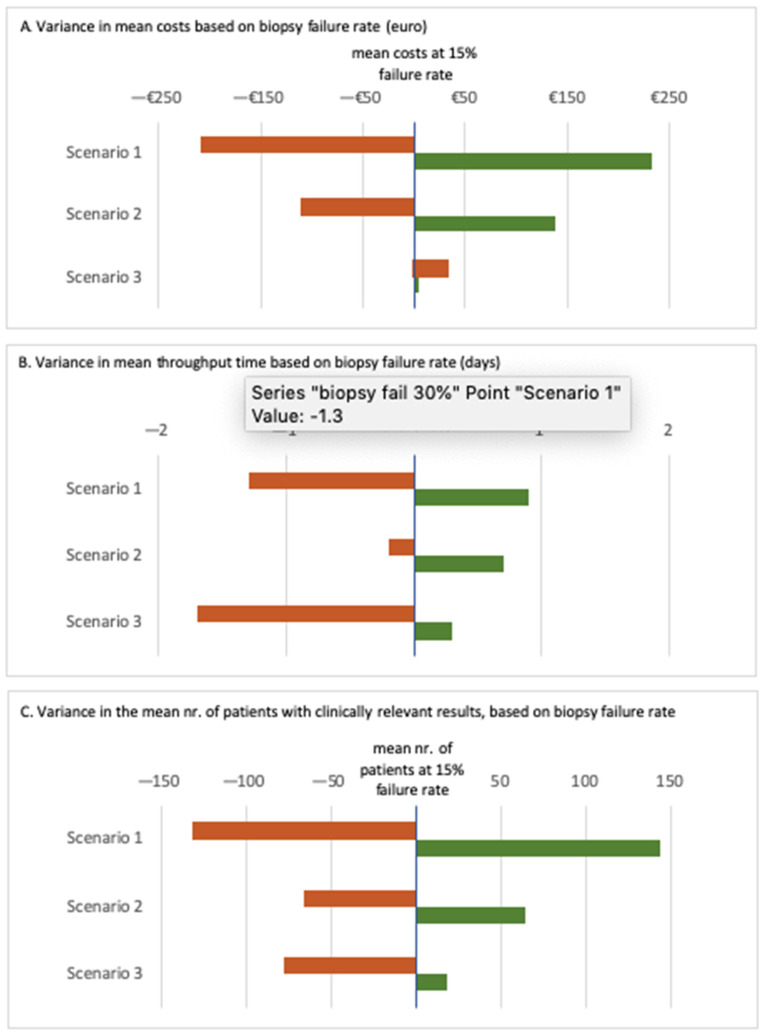
This figure shows the variance from the mean from the probabilistic sensitivity analysis (500 runs). The baseline point is (**A**) the mean of the costs for all patients with clinically relevant test results, (**B**) mean throughput time for all patients with clinically relevant test results, and (**C**) the mean number of patients with clinically relevant test results, with a tissue failure rate of 15%. The shown effect is for a tissue failure rate of 1% (green) to 30% (orange).

Figure 4 presents the results of the scenario analysis around the biopsy failure rate. A high tissue failure rate (30%) resulted in mean costs of €2096 in scenario 1, €3109 in scenario 2, and €2482 in scenario 3. In terms of time, a high tissue failure rate led to a median throughput time of 18, 9, and 17 days in scenarios 1, 2, and 3, respectively. In general, higher tissue failure rates (30%) led to a lower proportion of cases with clinically relevant test results: 71%, 86%, and 85% in scenarios 1, 2, and 3. 

In case of a low tissue failure rate, the mean costs of scenarios 1 and 2 were €2537 and €3357, while the mean cost of scenario 3 was €2452. The mean throughput time was 21, 10, and 19 days in scenarios 1, 2, and 3, respectively. The proportion of clinically relevant test results was high in all three scenarios, with low biopsy failure rates: 98%, 99%, and 95% in scenarios 1, 2, and 3, respectively (Figure 4).

This scenario analysis shows that a variance in tissue failure rate mostly effects mean total costs, mean throughput time and mean patients with clinically relevant test results in scenario 1. In case of high tissue failure rate, total costs, and throughput times decrease, but also at the costs of a low proportion of patients with relevant outcomes in the tissue -only scenario. When the tissue failure rate is low, costs and throughput times for analysis minorly increase, but the proportion of patients with relevant outcomes increases.

Most importantly, the outcomes of scenarios with cfDNA are less dependent on tissue failure rates.

## 4. Discussion

In this model-based analysis, we evaluated two possible scenarios for including cfDNA in molecular diagnostics, compared to tissue-based analysis only, for patients with stage IV NSCLC. Using a discrete event simulation, we analyzed the costs, throughput time, and proportion of patients with clinically relevant test results from each scenario, and compared it to a scenario resembling the current standard of care with molecular tissue diagnostics. 

The implementation of cfDNA for molecular diagnostics for stage IV NSCLC seems feasible, based on the model-based analysis on an organizational level. The implementation increased the mean extra cost by 900 euros (CrI €889–1004) per patient if offered to every mNSCLC patient (scenario 2), or led to a comparable mean cost per patient when offered after a failed biopsy attempt (scenario 3). When performing cfDNA for every patient (scenario 2), the median throughput time for molecular diagnostics was shorter (9 vs. 20 days). In the two scenarios with cfDNA, up to 93% of patients with molecular diagnostics based on cfDNA and tissue had a clinically relevant test result, compared to 85% in patients with tissue diagnostics only. This presumably leads to an improvement in access to treatment. 

Scenario 2, in which every patient gets cfDNA first, seemed to result in the shortest throughput time, but was also the costliest scenario. In this scenario, there was a reduction of biopsy attempts by 15% compared to scenario 1. Scenario 3, wherein cfDNA is implemented only after a failed biopsy, lead to a reduction of biopsy attempts by 8%; these all comprise second biopsies. The costs of these two scenarios were similar. Scenario 2 had the highest number of clinically relevant test results, including a high number of biomarkers. 

Scenario 3—tissue biopsy to start with, and a liquid biopsy if it fails—seems like a feasible alternative without extra costs. The throughput time and costs were comparable to those of scenario 1 (tissue biopsy only), but adding a liquid biopsy the percentage caused the proportion of clinically relevant test results to increase from 84% to 95%. However, the number of uncertain cases in the clinically relevant test results also increased: in scenario 3, the percentage of patients with an incomplete test result on cfDNA (KRAS, ERBB2, RET, or METe14 based on cfDNA but not PDL1) was still 11% of the total population.

This process-based analysis relies on several assumptions. It is important to note that both scenarios 2 and 3 rely on the assumption that oncologists are confident to start treatment based on an EGFR, BRAF, ALK, and ROS1 variant found with cfDNA. In the case of KRAS, ERBB2, RET, or METe14, only a small tissue sample for immunohistochemistry is required in scenario 2, with the assumption that there is no need for additional NGS in this scenario. In scenario 3, after one failed tissue attempt, we assumed that only cfDNA will be taken, and that there were no additional biopsies planned, even after negative or inconclusive results. One of the other assumptions was that the probability that a plasma analysis will technically succeed is 99.5%, based on the previous literature [11]. For the assumption of 15% tissue failure rates, we performed a scenario analysis since the tissue failure rates might vary between institutions. The results for these scenario analyses can be found in Figure 4. This figure shows that the results are quite robust across the spread in tissue failure rate of 0–30%.

A high specificity and high positive predictive value have been established for cfDNA in mNSCLC in the detection of targetable oncogenic variants. This was confirmed in clinical cohorts using targeted NGS-based assays [12,19,20,21]. Multiple experts in the field have suggested cfDNA as an option for genotyping in mNSCLC, since the genomic analysis of tumor biopsies is not always feasible [10,12,22]. Aggarwal et al. describe possible scenarios for the use of cfDNA in molecular diagnostics for patients with mNSCLC(10). Scenario 3 calculates the financial and clinical implications for cfDNA in the case of tissue not being available for genotyping. Scenario 2 reflects the advice to treat targetable drivers based on ctDNA results, and to pursue tumor genotyping if there are no mutations found in the cfDNA(10). Both scenarios are feasible, and our results might be valuable for making informed decisions. Potentially, the additional effect of cfDNA on clinically relevant test results could be higher than what we show in our simulation analysis. The yield of potentially targetable driver mutations from tissue molecular profiling was higher in the POPSTAR cohort (34.4%) than in others (20.5% and 21.3%) [10,11,12].

This model has several limitations. Importantly, the model does not take into account complications after biopsies, together with the associated patients’ reported quality of life, and associated costs, due to a lack of data. More registry data on the complications following tissue biopsies or biopsy attempts could provide more insight into the true institutional cost reduction. 

Additionally, this study reflects a process evaluation rather than a standard cost-effectiveness analysis, as results are not expressed in life years or quality-adjusted life years gained; such an analysis was outside the scope of this already comprehensive study. In this analysis, success rates are expressed in terms of the proportion of patients with valid test results, given the high failure rate in tissue diagnostics. On the one hand, health state utilities and disutility and associated costs for adverse events after biopsies would better inform about the consequences of tissue diagnostics. On the other hand, the subtle difference in costs of the diagnostic trajectory would be diluted by the costs of subsequent treatment in the total trajectory. The current analysis shows us the potential organizational consequences of the implementation of cfDNA in the diagnostic setting. In the LEMA study, the patients were treated according to the results of tissue diagnostics alone, so, unfortunately, no follow-up data were available for the cfDNA scenarios. Although it might theoretically be possible to model the consequences of biomarkers and subsequent treatments based on the literature, this would require numerous assumptions and introduce substantial uncertainty. Moreover, no guidelines are available for the acceptable costs for cancer diagnostics in relation to acquired (quality-adjusted) life-years. The proportion of patients with the identified biomarkers is higher in scenarios 2 and 3 (56%, 64%, and 62% for the three scenarios, respectively). This might give an indication of the opportunity to select patients for treatment with targeted immunotherapy, thereby increasing their life expectancy. 

## 5. Conclusions

In conclusion, this discrete event simulation analysis showed that adding cfDNA to the diagnostic workup is feasible and could increase the proportion of patients with a clinically relevant test result. That is under the condition that clinicians are willing to start treatment on a target found with cfDNA. Moreover, adding a cfDNA analysis reduced throughput times, especially if cfDNA was analyzed first. The addition of cfDNA led to marginal extra costs if secondary biopsy attempts were replaced with cfDNA. Finally, the implementation of cfDNA reduced the need for one or multiple tissue biopsies, presumably improving patients’ quality of life. This could be further explored in future prospective studies.

## Figures and Tables

**Table 1:** Deterministic results of one simulation run of 10,000 cases per scenario.

	Scenarios					
Outcomes of Deterministic Analysis	1. BIOPSY ALONE		1. BIOPSY ALONE		1. BIOPSY ALONE	
	Scenario 1	10,000 Patients	Scenario 2	10,000 Patients	Scenario 3	10,000 Patients
**Cost**						
Mean cost of the run in Euro (SD)	€2294	(€868)	€3350	(€1172)	€2443	(€592)
**Throughput time**						
Median throughput time of the run, in days (IQR)	20	(16–23)	10	(7–25)	18	(16–22)
**Test result**						
Nr. of patients with clinically relevant test result in the run (%)	8414	(84%)	9187	(92%)	8767	(88%)
*Complete test result (%)*	*8414*	*(84%)*	*8808*	*(88%)*	*7734*	*(77%)*
*Incomplete: cfDNA targets but no PDL1 status (%)*	*NA*	*-*	*379*	*(4%)*	*1033*	*(10%)*
Nr. of patients with no conclusive result (%)	1586	(16%)	813	(8%)	1233	(12%)

“Complete test result”: if a modeled patient has a negative or positive test result after complete molecular diagnostics, without any failures. This also implies to patients with all required test performed, but for whom all test results were negative for all biomarkers. “Incomplete”: patients with an incomplete profile of molecular diagnostics (e.g., cfDNA positive for KRAS but no biopsy/no PDL1 staining available). “No conclusive result”: If there is no test result due to failure of biopsies or failure of NGS. The grey, italic part is a subdevision of the above category.

**Table 2:** Results of the probabilistic sensitivity analysis: mean results of 1000 runs per scenario, presented with 95% quantiles.

Outcomes of Probabilistic Analysis	Scenarios								
	1. BIOPSY ALONE			2. cfDNA at Diagnosis, if EGFR, BRAF ALK, ROS1: Biopsy Cancelled			3. cfDNA if Biopsy Failed		
		1000 Runs		Scen. 2	1000 Runs		Scen. 3	1000 Runs	
		95% CrI			95% CrI			95% CrI	
	Mean	Upper	Lower	Mean	Upper	Lower	Mean	Upper	Lower
**Cost**									
Mean price of all runs, in Euro	€2304	€2067	€2507	€3218	€3071	€3396	€2448	€2382	€2506
**Throughput time**									
Mean throughput time of all runs, in days	20	17	23	9	7.0	10.6	19	16.2	21.7
**Clinically relevant test result**									
Mean nr. of patients with clinically relevant test result of all runs	8397 (84%)	6978	9428	9286 (93%)	8604	9759	9272 (93%)	8588	9889

Cost in exact Euro.

## Data Availability

Data and coding can be shared on request.

## Supplementary Materials

The following supporting information can be downloaded at: https://www.mdpi.com/article/10.3390/cancers14071783/s1. Figure S1: Cumulative mean values of cost, time and clinically relevant results, per scenario. 1 run with 20,000 cases, for determination of replications per run, Figure S2: Random draw of Dirichlet distributions for outcomes of tests, as an example of the draws used in the prob. sensitivity analysis, Figure S3: PSA outcomes: mean costs per run, Figure S4: PSA outcomes: median time per run, Figure S5: PSA outcomes: number of clinically relevant test results, Figure S6: Cumulative mean values of cost, time and clinically relevant results, per scenario. Plots for 600 runs with 10.000 cases for determination of total number of runs in probabilistic sensitivity analysis, Figure S7: Distribution of all input parameters used in the probabilistic sensitivity analysis, Table S1: Process-based cost calculations of diagnostic applications base, Table S2: Overview cost for ctDNA testing, Table S3: Input parameters for the model, table with mean probabilities, Table S4: Means and distributions for inter-patient variance in both the base case and PSA model, Table S5: PSA outcomes: mean number of patients with 0, 1 or 2 biopsies, listed tabularly per scenario.
