# Supplementary material for: Cell-Free DNA at Diagnosis for Stage IV Non-Small Cell Lung Cancer: Costs, Time to Diagnosis and Clinical Relevance
Source: Cancers (Basel). 2022 Mar 31;14(7):1783. doi: 10.3390/cancers14071783 (PMC8997553; doi:10.3390/cancers14071783)
Supplement: Supplementary file 1 [file cancers-14-01783-s001.zip › cancers-1629959-supplementary.pdf]

<u>Supplementary files</u>

Cell-free DNA at diagnosis for stage IV non-small cell lung cancer: costs, time to diagnosis and clinical relevance

S.N. Koole[1], D.C.L. Vessies[2], M.M.F. Schuurbiers[3], A. Kramer[4], R.D. Schouten[5], K. Degeling[6], L.J.W. Bosch[7], M.M. van den Heuvel[3], W.H. van Harten[1,8], D. van den Broek[9], K. Monkhorst*[7], V.P. Retèl*[1,8]

1. Division of Psychosocial Research and Epidemiology, The Netherlands Cancer Institute, Amsterdam, The Netherlands
2. Department of Laboratory Medicine, Netherlands Cancer Institute, Amsterdam, the Netherlands.
3. Department of Pulmonary Diseases, Radboud University Medical Centre, Nijmegen, The Netherlands
4. Department of Epidemiology and DataData Science, Amsterdam University Medical Centers (location VUmc), Amsterdam, The Netherlands
5. Department of pulmonology, The Netherlands Cancer Institute, Amsterdam, The Netherlands
6. Cancer Health Services Research, Centre for Health Policy & Centre for Cancer Research, Faculty of Medi-cine, Dentistry and Health Sciences, The University of Melbourne, Melbourne, Australia
7. Department of Pathology, Netherlands Cancer Institute, Amsterdam, The Netherlands
8. Department of Health Technology and Services Research University of Twente, Enschede The Nether-lands.
9. Department of Clinical Chemistry, The Netherlands Cancer Institute, Amsterdam, the Netherlands.

* Correspondence: dr. V.P. Retèl, v.retel@nki.nl;

Supplementary Table S1. Process-based cost calculations of diagnostic applications based on the standard case perspective. Adapted from an earlier publication of Pasmans et al.(1)[a]

	Techniques																	
	IHC		FISH	Pyro seq	HRM		Sanger				NGS			Cobas	Biocartis			
Additional equipment	Light microscope, Leica	Light microscope, Leica	Hybridizer (DAKO, Agilent)								Ion Chef + PCR apparatus	Ion Chef + PCR apparatus	Ion Chef + PCR apparatus		Idylla console	Idylla console	Idylla console	Idylla console
Life cycle additional equipment (years)	10	10	5								5	5	5		10	10	10	10
Capacity additional equipment per year	7020	7020	1498								666	1331	1331		624	624	624	624
Platform	Ventana, Roche	Ventana, Roche	Fluorescence microscope, Leica	Pyromark Q24, Qiagen	LC480, Roche	LC480, Roche	Applied Biosystems, ThemoFisher	Applied Biosystems, ThemoFisher	Applied Biosystems, ThemoFisher	Applied Biosystems, ThemoFisher	IonTorrent PGM, ThermoFisher	IonTorrent PGM, ThermoFisher	MiSeq, Illumina	Cobas, Roche	Idylla, Biocartis	Idylla, Biocartis	Idylla, Biocartis	Idylla, Biocartis
Platform type	ALK, ROS1	PD-1, PD-L1	ALK, ROS1, RET	EGFR+KRAS hotspots (6 amplicons)	EGFR+KRAS+BRAF hotspots (7-8 amplicons)	BRAF+NRAS (3 amplicons)	ABI3500 (10 amplicons: EGFR, KRAS, BRAF, ERBB2, MET)	ABI3500 (3 amplicons: BRAF, NRAS)	ABI3500 (6 amplicons: KRAS, NRAS, BRAF)	ABI3500 (9 amplicons: KIT, PDGFRA, BRAF)	PGM: 316 chip, cancerhotspot panel v2	PGM: 318 chip, cancerhotspot panel v2	MiSeq: 2x150 bp micro v2 kit, cancer hotspot panel v2	BRAF	BRAF	EGFR	KRAS	BRAF+NRAS
Life cycle platform (years)	10	10	10	5	10	10	7	7	7	7	5	5	5	10	5	5	5	5
Utilization	30%	30%	24%	8%	56%	28%	54%	54%	54%	54%	32%	32%	32%	0.3%	60%	60%	60%	60%
Actual annual throughput	7020	7020	1498	666	1747	1747	18870	18870	18870	18870	666	1331	1331	117	624	624	624	624
**Capital costs**																		
Additional equipment initial costs[b]	€ 50,000.00	€ 50,000.00	€ 6,679.00	€ 0.00	€ 0.00	€ 0.00	€ 0.00	€ 0.00	€ 0.00	€ 0.00	€ 9,000.00	€ 9,000.00	€ 9,000.00	€ 0.00	€ 5,000.00	€ 5,000.00	€ 5,000.00	€ 5,000.00
Life cycle additional equipment (years)	10	10	5	1	1	1	1	1	1	1	5	5	5	1	10	10	10	10
Platform initial costs[b]	€ 15,000.00	€ 15,000.00	€ 70,000.00	€ 70,944.00	€ 65,000.00	€ 65,000.00	€ 136,500.00	€ 136,500.00	€ 136,500.00	€ 136,500.00	€ 61,897.00	€ 61,897.00	€ 95,811.00	€ 64,060.37	€ 45,000.00	€ 45,000.00	€ 45,000.00	€ 45,000.00
Life cycle platform (years)	10	10	10	5	10	10	7	7	7	7	5	5	5	10	5	5	5	5
Interest rate (0-1)	0.05	0.05	0.05	0.05	0.05	0.05	0.05	0.05	0.05	0.05	0.05	0.05	0.05	0.05	0.05	0.05	0.05	0.05
Annuity factor additional equipment	7.91	7.91	4.39								4.39	4.39	4.39		7.91	7.91	7.91	7.91
Annuity factor platform	7.91	7.91	7.91	4.39	7.91	7.91	5.89	5.89	5.89	5.89	4.39	4.39	4.39	7.91	4.39	4.39	4.39	4.39
Annual capital costs additional equipment[c]	€ 6,318.94	€ 6,318.94	€ 1,521.42	€ 0.00	€ 0.00	€ 0.00	€ 0.00	€ 0.00	€ 0.00	€ 0.00	€ 2,050.12	€ 2,050.12	€ 2,050.12	€ 0.00	€ 631.89	€ 631.89	€ 631.89	€ 631.89
Annual capital costs platform[c]	€ 1,895.68	€ 1,895.68	€ 8,846.52	€ 16,160.45	€ 8,214.62	€ 8,214.62	€ 23,164.25	€ 23,164.25	€ 23,164.25	€ 23,164.25	€ 14,099.62	€ 14,099.62	€ 21,824.94	€ 8,095.87	€ 10,250.62	€ 10,250.62	€ 10,250.62	€ 10,250.62
Annual capital costs additional equipment (sample preparation platform)																		
Annual capital costs sequencing per 30x cov																		
VAT (21%) for equipment	€ 13,650.00	€ 13,650.00	€ 16,102.59	€ 14,898.24	€ 13,650.00	€ 13,650.00	€ 28,665.00	€ 28,665.00	€ 28,665.00	€ 28,665.00	€ 14,888.37	€ 14,888.37	€ 22,010.31	€ 13,452.68	€ 10,500.00	€ 10,500.00	€ 10,500.00	€ 10,500.00
Capital costs per sample or per tumor normal[d]	**€ 3.11**	**€ 3.11**	**€ 17.68**	**€ 46.66**	**€ 12.51**	**€ 12.51**	**€ 2.75**	**€ 2.75**	**€ 2.75**	**€ 2.75**	**€ 46.63**	**€ 23.32**	**€ 34.47**	**€ 184.18**	**€ 34.27**	**€ 34.27**	**€ 34.27**	**€ 34.27**
**Maintenance costs**																		
Annual maintenance costs additional equipment (first year)[e]	€ 0.00	€ 0.00	€ 0.00	€ 0.00	€ 0.00	€ 0.00	€ 0.00	€ 0.00	€ 0.00	€ 0.00	€ 0.00	€ 0.00	€ 0.00	€ 0.00	€ 0.00	€ 0.00	€ 0.00	€ 0.00
Annual maintenance costs additional equipment (other years)	€ 5,000.00	€ 5,000.00	€ 200.00	€ 0.00	€ 0.00	€ 0.00	€ 0.00	€ 0.00	€ 0.00	€ 0.00	€ 900.00	€ 900.00	€ 900.00	€ 0.00	€ 500.00	€ 500.00	€ 500.00	€ 500.00
Annual maintenance costs platform (first year)[e]	€ 0.00	€ 0.00	€ 0.00	€ 0.00	€ 0.00	€ 0.00	€ 0.00	€ 0.00	€ 0.00	€ 0.00	€ 0.00	€ 0.00	€ 0.00	€ 0.00	€ 0.00	€ 0.00	€ 0.00	€ 0.00
Annual maintenance costs platform (other years)	€ 500.00	€ 500.00	€ 1,000.00	€ 6,500.00	€ 3,148.00	€ 3,148.00	€ 3,655.00	€ 3,655.00	€ 3,655.00	€ 3,655.00	€ 6,100.00	€ 6,100.00	€ 11,867.00	€ 5,200.00	€ 4,000.00	€ 4,000.00	€ 4,000.00	€ 4,000.00
Annual maintenance costs	€ 4,950.00	€ 4,950.00	€ 1,060.00	€ 5,200.00	€ 2,833.20	€ 2,833.20	€ 3,132.86	€ 3,132.86	€ 3,132.86	€ 3,132.86	€ 5,600.00	€ 5,600.00	€ 10,213.60	€ 4,680.00	€ 3,650.00	€ 3,650.00	€ 3,650.00	€ 3,650.00
Annual maintenance costs additional equipment																		
Annual maintenance costs sequencing per 30xcov																		
Maintenance costs per sample or per tumor normal[d]	**€ 0.71**	**€ 0.71**	**€ 0.71**	**€ 7.81**	**€ 1.62**	**€ 1.62**	**€ 0.17**	**€ 0.17**	**€ 0.17**	**€ 0.17**	**€ 8.41**	**€ 4.21**	**€ 7.67**	**€ 17.14**	**€ 5.85**	**€ 5.85**	**€ 5.85**	**€ 5.85**
**Software costs**																		
Aqcuisition software costs[b]							€ 2,000.00	€ 2,000.00	€ 2,000.00	€ 2,000.00	€ 20,000.00	€ 20,000.00	€ 20,000.00					
Life cycle software (years)							1	1	1	1	10	10	10					
Annual software management / maintenance costs[f]																		
Annual software costs	€ 0.00	€ 0.00	€ 0.00	€ 0.00	€ 0.00	€ 0.00	€ 2,000.00	€ 2,000.00	€ 2,000.00	€ 2,000.00	€ 3,500.00	€ 3,500.00	€ 3,500.00	€ 0.00	€ 0.00	€ 0.00	€ 0.00	€ 0.00
Software costs per sample or per tumor normal[d] [f]	**€ 0.00**	**€ 0.00**	**€ 0.00**	**€ 0.00**	**€ 0.00**	**€ 0.00**	**€ 0.11**	**€ 0.11**	**€ 0.11**	**€ 0.11**	**€ 5.26**	**€ 2.63**	**€ 2.63**	**€ 0.00**	**€ 0.00**	**€ 0.00**	**€ 0.00**	**€ 0.00**
**Operational costs**																		
Sample preparation and quality control consumables per 30x cov[b]																		
Sample preparation and quality control consumables per sample[b]	€ 69.10	€ 60.96	€ 79.60	€ 319.05	€ 46.13	€ 23.07	€ 19.30	€ 5.79	€ 11.58	€ 17.37	€ 106.48	€ 106.48	€ 140.57	€ 251.74	€ 140.00	€ 250.00	€ 190.00	€ 250.00
Sequencing consumables per 30x cov[b]																		
Consumables per sample[b]					€ 3.57	€ 3.57					€ 120.29	€ 81.19	€ 33.75	€ 7.78				
Data processing (per CPU hour / IT infra per tumor normal)[g]	€ 0.10	€ 0.10	€ 0.10	€ 0.10	€ 0.10	€ 0.10	€ 0.10	€ 0.10	€ 0.10	€ 0.10	€ 0.01	€ 0.01	€ 0.01	€ 0.10	€ 0.10	€ 0.10	€ 0.10	€ 0.10
Data storage (per GB storage per year)[g]	€ 0.01	€ 0.01	€ 0.01	€ 0.01	€ 0.01	€ 0.01	€ 0.01	€ 0.01	€ 0.01	€ 0.01	€ 0.05	€ 0.05	€ 0.05	€ 0.01	€ 0.01	€ 0.01	€ 0.01	€ 0.01
Personnel sample preparation and primary data analysis per sample[h]	€ 20.59	€ 20.59	€ 32.71	€ 37.14	€ 28.58	€ 28.58	€ 37.38	€ 37.38	€ 37.38	€ 37.38	€ 50.82	€ 42.08	€ 42.08	€ 33.02	€ 28.05	€ 28.05	€ 28.05	€ 28.05
Personnel data interpretation and report per sample[i]	€ 10.21	€ 10.21	€ 14.43	€ 16.98	€ 12.90	€ 12.90	€ 12.90	€ 12.90	€ 12.90	€ 12.90	€ 14.26	€ 14.26	€ 14.26	€ 12.90	€ 16.29	€ 16.29	€ 16.29	€ 16.29
VAT (21%) for equipment	€ 14.51	€ 12.80	€ 16.72	€ 67.00	€ 10.44	€ 5.59	€ 4.05	€ 1.22	€ 2.43	€ 3.65	€ 47.62	€ 39.41	€ 36.61	€ 54.50	€ 29.40	€ 52.50	€ 39.90	€ 52.50
Operational costs per sample or per tumor normal[d]	**€ 114.52**	**€ 104.67**	**€ 143.57**	**€ 440.28**	**€ 101.73**	**€ 73.83**	**€ 73.74**	**€ 57.40**	**€ 64.40**	**€ 71.41**	**€ 339.53**	**€ 283.48**	**€ 267.33**	**€ 360.04**	**€ 213.85**	**€ 346.95**	**€ 274.35**	**€ 346.95**
**Total costs per cancer patient[j]**	**€ 118.34**	**€ 108.49**	**€ 161.95**	**€ 494.75**	**€ 115.87**	**€ 87.96**	**€ 76.76**	**€ 60.42**	**€ 67.42**	**€ 74.43**	**€ 399.84**	**€ 313.63**	**€ 312.10**	**€ 561.36**	**€ 253.97**	**€ 387.07**	**€ 314.47**	**€ 387.07**
**INCLUDING OVERHEAD**	**€ 170.41**	**€ 156.23**	**€ 233.21**	**€ 712.44**	**€ 166.85**	**€ 126.67**	**€ 110.54**	**€ 87.00**	**€ 97.09**	**€ 107.18**	**€ 575.77**	**€ 451.63**	**€ 449.42**	**€ 808.36**	**€ 365.71**	**€ 557.38**	**€ 452.83**	**€ 557.38**

[a] The standard case perspective implies that an average Dutch lab practice was assumed and suppliers' standard list prices were used, in respect of all techniques.

[b] The cost of the platforms, software and consumables all include Value Added Taxes (21% VAT).

[c] For the standard diagnostic techniques, life cycles varying between 5 to 10 years, annuity factors ranging between 4,39 to 7,91, and an interest rate of 4,5 % are maintained for both types of equipment (if applicable). The annual capital costs of the additional equipment (sample preparation platform) and the sequencing platform for WGS are calculated by taking into account a life cycle of 10 and 5 years, an annuity factor of 8,11 and 4,45, respectively, and an interest rate of 4 %.

[d] The capital, maintenance and operational costs per sample calculations are based on 2 times sample preparation and 4 times genome sequencing (90x coverage tumor and 30x coverage blood) for WGS. For WGS application, two samples are needed (tumor biopsy and blood) to do the analysis whereas for the standard used techniques 1 sample (tumor) suffices.

[e] During the first year no maintenance costs occur as the platforms have a warranty for the first year.

[f] Software management / maintenance incorporates daily supervision and maintenance of the pipeline for WGS. It takes up 0.2 FTE (of a 40-hour working week) for a bioinformatics technician with a gross hourly salary of € 50.

[g] Data processing and data storage are outsourced for WGS. The cost of data processing covers the complete analysis of raw data to BAM file, VCF file and patient report. The cost of data storage is estimated based on hot storage of the BAM file, VCF file and patient report for 6 months (€ 4 per month per 200 GB).

[h] The sample preparation and primary data analysis is done by a laboratory technician (gross hourly salary of € 22) and bioinformatics technician (gross hourly salary of € 29) for the standard techniques. For WGS this is performed by a laboratory technician (gross hourly salary of €25). Sample and report administration is incorporated for all techniques.

[I] The data interpretation and report per sample is done by a clinical molecular biologist (gross hourly salary of € 41) and pathologist (gross hourly salary of € 61) for the standard techniques. For WGS this is performed by a clinical molecular biologist and a bioinformatics technician, both with a gross hourly salary of €50.

[J] The total cost per cancer patient represents a total cost per target gene separately for IHC (ALK, ROS1) and FISH (ALK, ROS1, RET). A combined total cost per cancer patient of the specified target genes per technique is given for Pyro seq, HRM (EGFR + KRAS + BRAF; BRAF + NRAS) and Biocartis (BRAF + NRAS)), and for Sanger (10, 3, 6, 9 amplicons) and NGS hotspot panels.

Supplementary Table S2. Overview cost for ctDNA testing:

	**Personnel costs p/s**	**Material costs p/s**	**Equipment costs p/s**	**Tariff p/s**	**Total cost p/s**
1. Obtain sample in In cell-stabilizing tube	€ 0,00	€ 26,14	€ 0,00	€ 13,70	€ 39,84
2. Transport sample	€ 2,79	€ 3,64	€ 0,00		€ 6,43
3. Process sample	€ 11,17	€ 3,25	€ 0,00		€ 14,43
4. DNA isolation with QiaSymphony SP	€ 2,99	€ 48,36	€ 11,33		€ 62,68
5. ctDNA analysis with Avenio ctDNA Targeted Kit (Roche) + NextSeq 550 (Illumina)	€ 29,72	€ 878,92	€ 46,34		€ 954,98
6. Report results	€ 9,50	€ 0,00	€ 0,00		€ 9,50
Total:	€ 56,17	€ 960,30	€ 57,67	€ 13,70	**€ 1.087,85**
Failure costs				0,5%	€ 5,09
Overhead + facility costs				44%	€ 480,89
					**€ 1.573,83**

Data sources used were hospital-specific tariffs, time measurements, internal invoices, published list prices, and expert input.

Main assumptions for cost ctDNA:

- Activity based costing method, including the costs for personnel, materials, and equipment.
- Cost price is based on analyzing 12 samples per week (in one run).
- For failure costs and overhead and facility costs we used a mark-up
  - 44% mark-up for overhead and facility costs. We assumed that these costs covered the costs for multi-purpose equipment and all indirect costs.
  - 0,5% failure rate (include the costs for re-performing the ctDNA analysis with the same sample for 0,5% of the samples).
- Cost for material and equipment costs includes 21% VAT.
- Equipment 33% utilization

Supplementary Table S3. Input parameters for the model, table with mean probabilities

	Mean probability measured from trial data (base-case)	S.E.	Distribution type (PSA)			Used in:	Assumption S.E.
**PLASMA**							
probability that plasma retrieval succeeds*	99.5%		-			Scen 2+3	
probability of finding:							
* EGFR with cfDNA	0.063	0.006	Dirichlet			Scen 2+3	10%
* ROS1 with cfDNA	0.005	0.001	Dirichlet			Scen 2+3	10%
* BRAF with cfDNA	0.024	0.002	Dirichlet			Scen 2+3	10%
* ALK with cfDNA	0.015	0.002	Dirichlet			Scen 2+3	10%
* METe14 with cfDNA	0.015	0.002	Dirichlet			Scen 2+3	10%
* KRAS with cfDNA	0.344	0.034	Dirichlet			Scen 2+3	10%
* MET with cfDNA	0.029	0.003	Dirichlet			Scen 3	10%
* RET with cfDNA	0.005	0.001	Dirichlet			Scen 3	10%
* HER2 with cfDNA	0.015	0.002	Dirichlet			Scen 3	10%
* NRAS with cfDNA	0.010	0.001	Dirichlet			Scen 3	10%
* rest	0.117	0.012	Dirichlet			Scen 2+3	10%
**TISSUE**							
probability that a biopsy fails	15%	0.064	Beta	5	26	Scen 1+2+3	
probability that 2 biopsies are needed for mol. Analysis	12%	0.053	Beta	4	32	Scen 1+2+3	
probability that tissue NGS succeeds after successful biopsy*	99%	0.005	Beta	399	4	Scen 1+2+3	
probability that a biopsy fails in scenario 3	25%	0.094	Beta	5	15	Scen 1+2+3	

probability that a "simple biopsy" succeeds	90%	0.045	Beta	39	4	Scen 1+2+3	
probability of finding:							
- KRAS with biopsy	0.440	0.044	Dirichlet			Scen 1+3	10%
-- pdl1 high after KRAS	*0.525*	0.053	Dirichlet			Scen 1+3	10%
-- pdl1 low after KRAS	*0.475*	0.048	Dirichlet			Scen 1+3	10%
- EGFR with biopsy	0.093	0.009	Dirichlet			Scen 1+3	10%
- BRAF with biopsy	0.033	0.003	Dirichlet			Scen 1+3	10%
- ALK with biopsy	0.032	0.003	Dirichlet			Scen 1+3	10%
- ROS1 with biopsy	0.032	0.003	Dirichlet			Scen 1+3	10%
- RET with biopsy	0.011	0.001	Dirichlet			Scen 1+3	10%
- METe14 with biopsy	0.022	0.002	Dirichlet			Scen 1+3	10%
- HER2 with biopsy	0.032	0.003	Dirichlet			Scen 1+3	10%
- NRAS with biopsy	0.097	0.010	Dirichlet			Scen 1+3	10%
- WT and pdl1 high	0.256	0.026	Dirichlet			Scen 1+3	10%
* WT and pdl1 low	0.527	0.053	Dirichlet			Scen 1+3	10%
**TISSUE after EGFR/ROS1/BRAF/ALK/METe14 cfDNA discoverage (N=106)**							
- EGFR with biopsy	0.039	0.004	Dirichlet			Scen 2	10%
- BRAF with biopsy	0.010	0.001	Dirichlet			Scen 2	10%
- KRAS with biopsy	0.108	0.011	Dirichlet			Scen 2	10%
-- pdl1 high after KRAS	0.455	0.046	Dirichlet			Scen 2	10%
- pdl1 low after KRAS	0.545	0.055	Dirichlet			Scen 2	10%
- ALK with biopsy	0	0.000	Dirichlet			Scen 2	10%
- ROS1 with biopsy	0.022	0.002	Dirichlet			Scen 2	10%
- RET with biopsy	0.077	0.008	Dirichlet			Scen 2	10%
- METe14 with biopsy	0	0.000	Dirichlet			Scen 2	10%
- HER2 with biopsy	0	0.000	Dirichlet			Scen 2	10%
- MET/NRAS with biopsy	0.121	0.012	Dirichlet			Scen 2	10%
- WT and pdl1h	0.264	0.026	Dirichlet			Scen 2	10%

- WT and pdl1 low	0.538	0.054	Dirichlet			Scen 2	10%
**Means and distributions for PSA runs**							
**TIME ESTIMATION***							
time for cfDNA	7.5	0.8	gamma	100.0	0.08	Scen 2+3	10%
time for biopsy	7	0.7	gamma	100.0	0.07	Scen 1+2+3	10%
time for tissue NGS + 3 IHC	11	1.1	gamma	100.0	0.11	Scen 1+2+3	10%
time for FISH/IHC	5	0.5	gamma	100.0	0.05	Scen 1+2+3	10%
time for biopsy after ctDNA-neg	2	0.2	gamma	100.0	0.02	Scen 2	10%
**COST ESTIMATION***							
tissue (biopsy)	€ 1,000	€ 100.0	gamma	100.0	10.00	Scen 1+2+3	10%
blood cfDNA	€ 1,574	€ 157.4	gamma	100.0	15.74	Scen 2+3	10%
tissue preparation costs	€ 72	€ 7.2	gamma	100.0	0.72	Scen 1+2+3	10%
tissue NGS	€ 556	€ 55.6	gamma	100.0	5.56	Scen 1+2+3	10%
PDL1 IHC	€ 156	€ 15.6	gamma	100.0	1.56	Scen 1+2+3	10%
PDL1 IHC + tissueprep	€ 228	€ 22.8	gamma	100.0	2.28	Scen 1+2+3	10%
tissue prep + 3 IHC	€ 384	€ 38.4	gamma	100.0	3.84	Scen 1+2+3	10%
FISH	€ 233	€ 23.3	gamma	100.0	2.33	Scen 1+2+3	10%
rtPCR	€ 100	€ 10.0	gamma	100.0	1.00	Scen 1+2+3	10%

*Not based on trial data. Estimated based on expert elicitations or measurements from literature (1) and the pathology department.
Abbreviations: S.E., standard error.

Supplementary Table S4. Means and distributions for inter-patient variance in both the base case and PSA model

	Mean probability measured from trial data (base-case)	S.E.	Distribution type (base-case)	alpha	beta	Used in:	Assumption SD
**TIME ESTIMATION**							
time for cfDNA	7.5	1.3	gamma	34.6	0.22	Scen 2+3	17%
time for biopsy	7	1.2	gamma	34.6	0.20	Scen 1+2+3	17%
time for tissue NGS + 3 IHC	11	1.9	gamma	34.6	0.32	Scen 1+2+3	17%
time for FISH/IHC	5	0.9	gamma	34.6	0.14	Scen 1+2+3	17%
time for biopsy after ctDNA-neg	2	0.3	gamma	34.6	0.06	Scen 2	17%
**COST ESTIMATION**							
tissue (biopsy)	€ 1,000	€ 170	gamma	34.6	28.90	Scen 1+2+3	17%
blood cfDNA	€ 1,574	€ 268	gamma	34.6	45.49	Scen 2+3	17%
tissue preparation costs	€ 72	€ 12	gamma	34.6	2.07	Scen 1+2+3	17%
tissue NGS	€ 556	€ 95	gamma	34.6	16.08	Scen 1+2+3	17%
PDL1 IHC	€ 156	€ 27	gamma	34.6	4.51	Scen 1+2+3	17%
PDL1 IHC + tissueprep	€ 228	€ 39	gamma	34.6	6.59	Scen 1+2+3	17%
tissue prep + 3 IHC	€ 384	€ 65	gamma	34.6	11.10	Scen 1+2+3	17%
FISH	€ 233	€ 40	gamma	34.6	6.74	Scen 1+2+3	17%
rtPCR	€ 100	€ 17	gamma	34.6	2.90	Scen 1+2+3	17%

Abbreviations: S.E., standard error.

Supplementary Table S5. PSA outcomes: mean number of patients with 0, 1 or 2 biopsies, listed tabularly per scenario.

	**BIOPSY ALONE**				**cfDNA at diagnosis, if EGFR, BRAF ALK, ROS1: biopsy cancelled**				**cfDNA if biopsy failed**			
	**Scenario 1**		**500 runs 95%CrI**		**Scenario 2**		**500 runs 95%CrI**		**Scenario 3**		**500 runs 95%CrI**	
	**Mean**	**%**	**Upper**	**Lower**	**Mean**	**%**	**Upper**	**Lower**	**Mean**		**Upper**	**Lower**
**No. of biopsy attempts**					1235	12%	786	1684	0	0%	0	0
0 attempts	0	0%	0	0	681	7%	440	1348	1660	17%	1208	3107
1 attempt, no biopsy	1525	15%	1008	2920	7654	77%	6812	8422	8340	83%	6893	9467
1 biopsy	7523	75%	6081	8843	430	4%	117	921	0	0%	0	0
2 biopsies	951	10%	300	2005	1235	12%	786	1684	0	0%	0	0
**Mean number of biopsy attempts per patient**	**1.10**				**0.92**				**1.00**			

Supplementary Figure S1. Cumulative mean values of cost, time and clinically relevant results, per scenario. 1 run with 20,000 cases, for determination of replications per run.

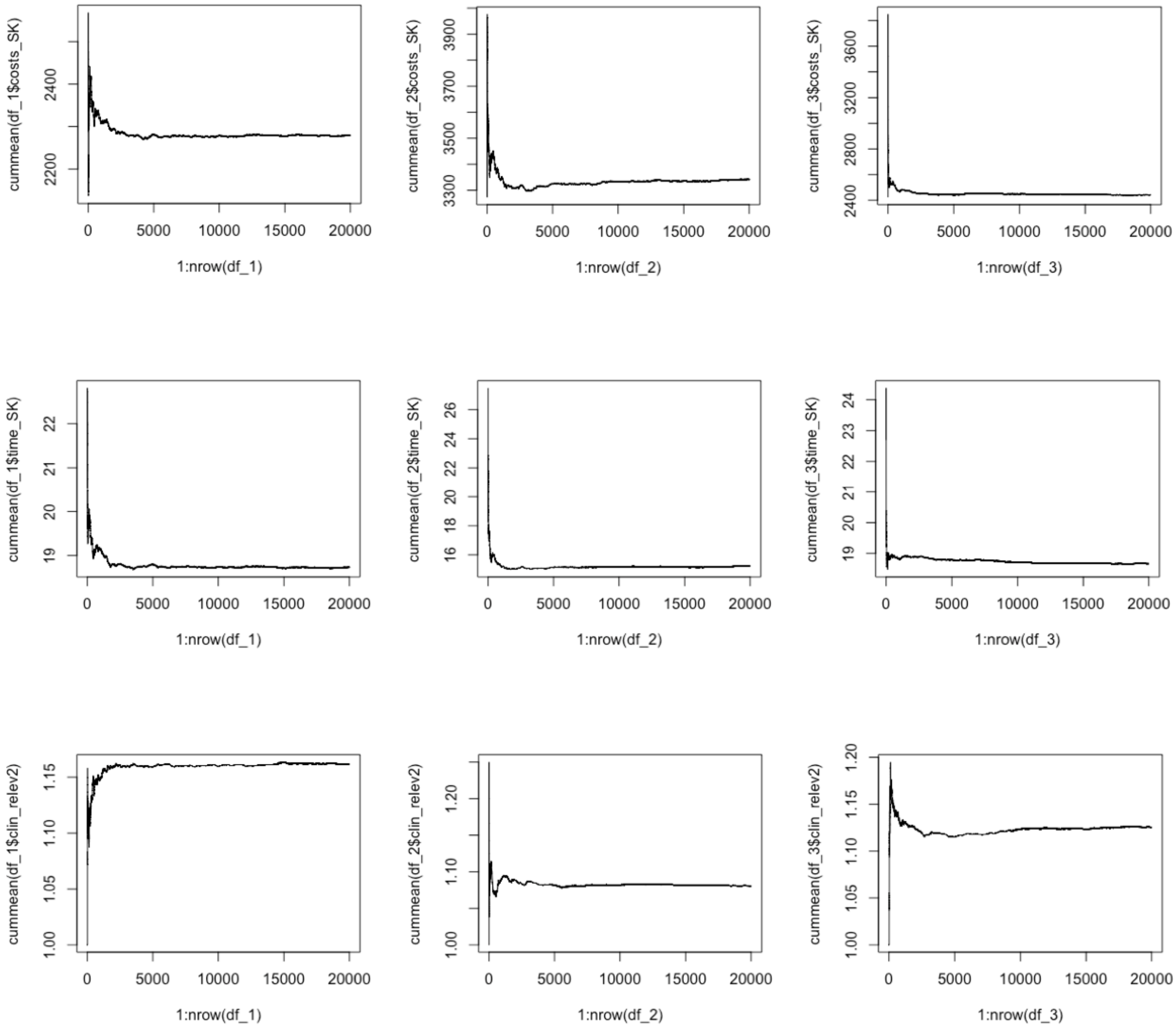

Df_1, scenario 1; Df_2, scenario 2; Df_3, scenario 3; cost, mean total cost per run; time, mean total throughput time; clin_relev2, clinically relevant test results.

Supplementary Figure S2: Random draw of Dirichlet distributions for outcomes of tests, as an example of the draws used in the prob. sensitivity analysis

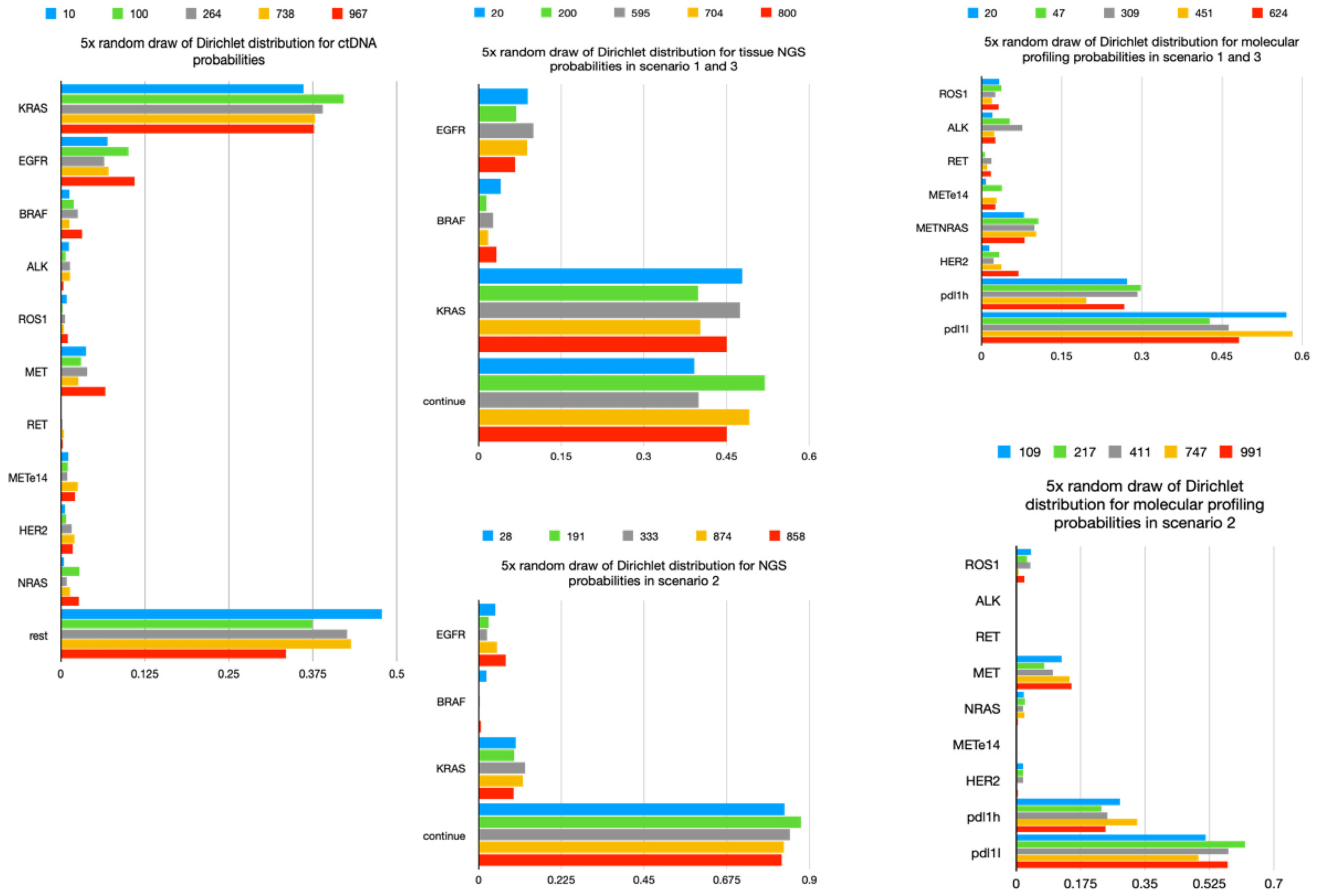

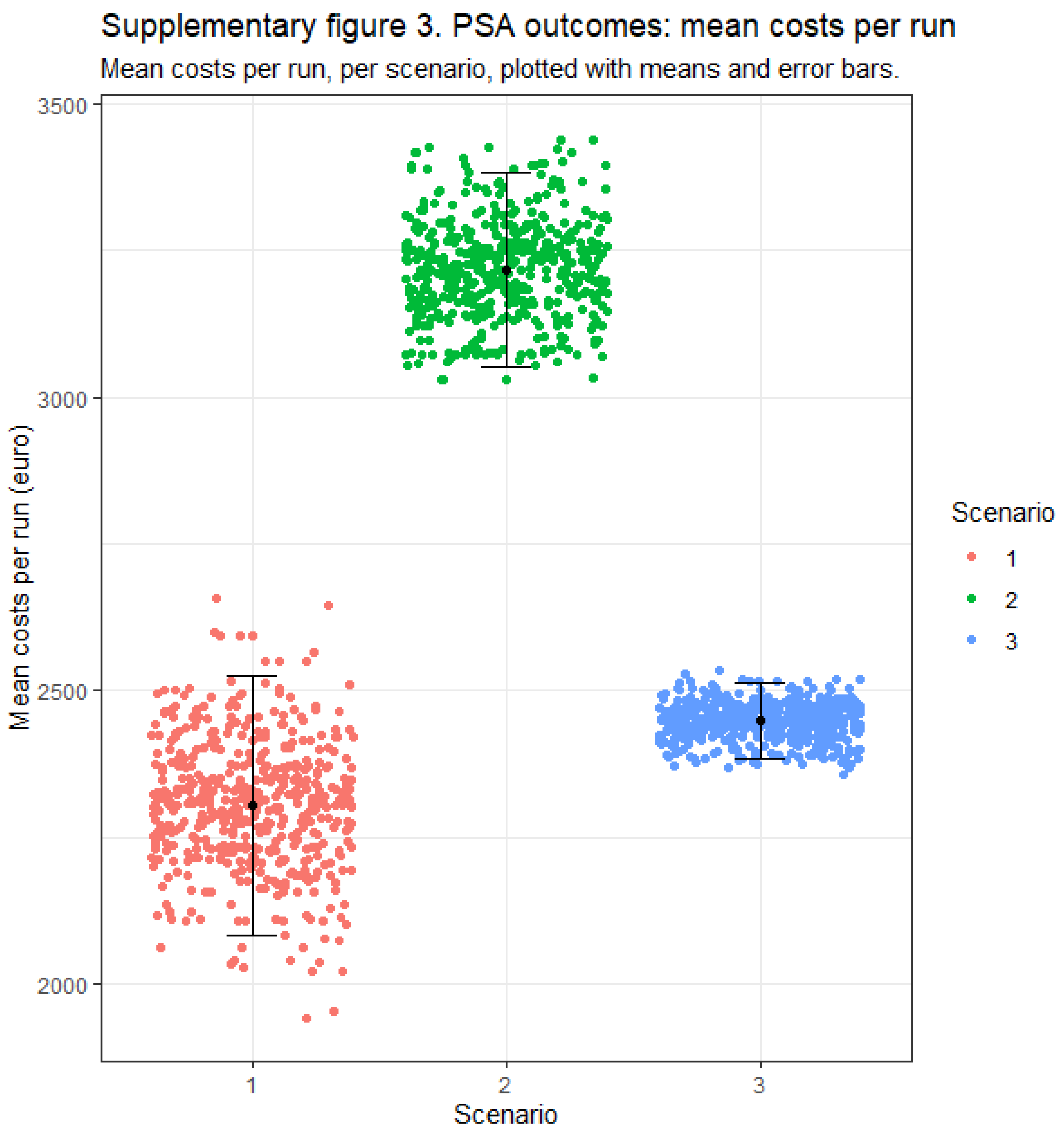

Supplementary Figure S3

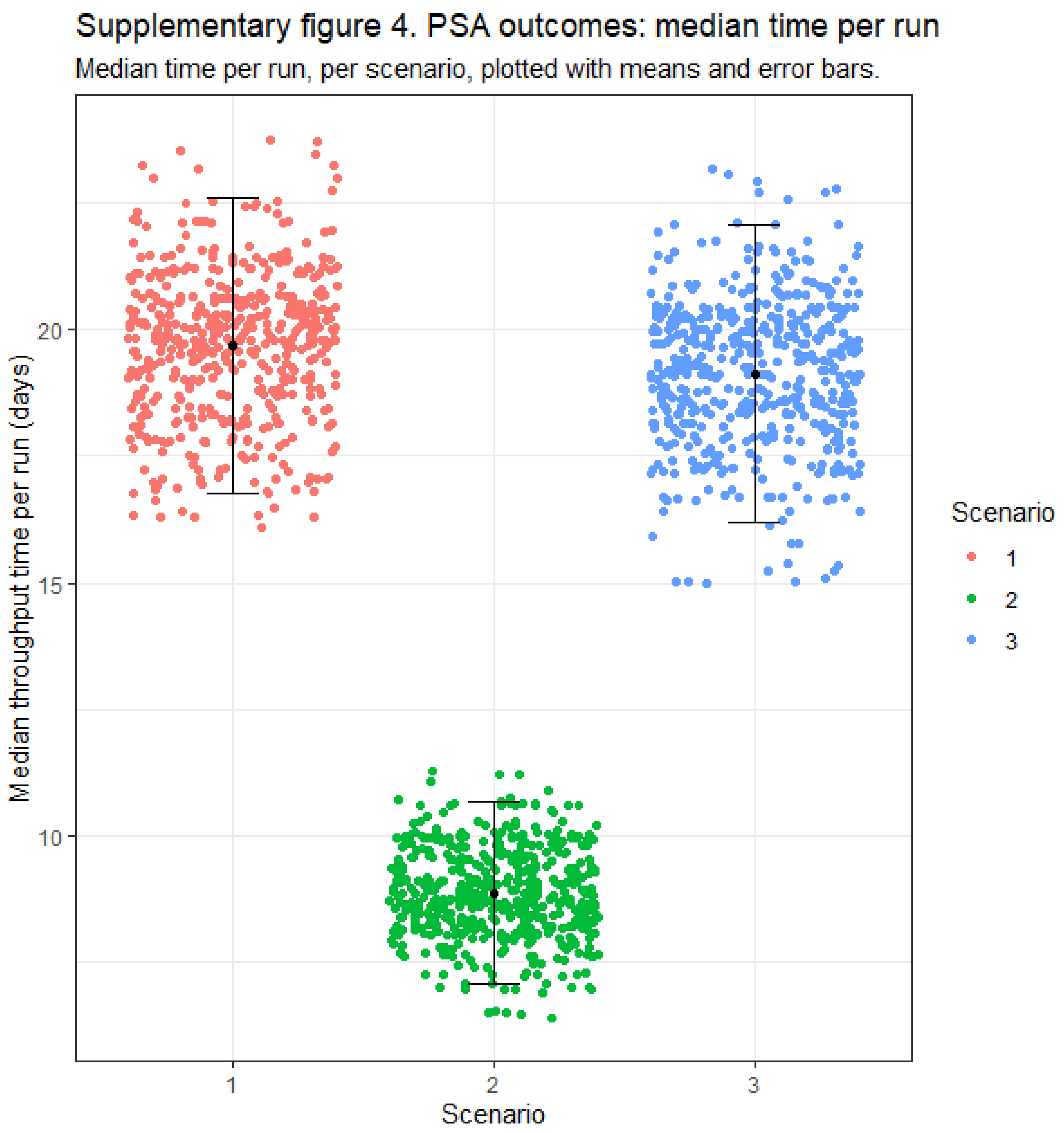

Supplementary Figure S4

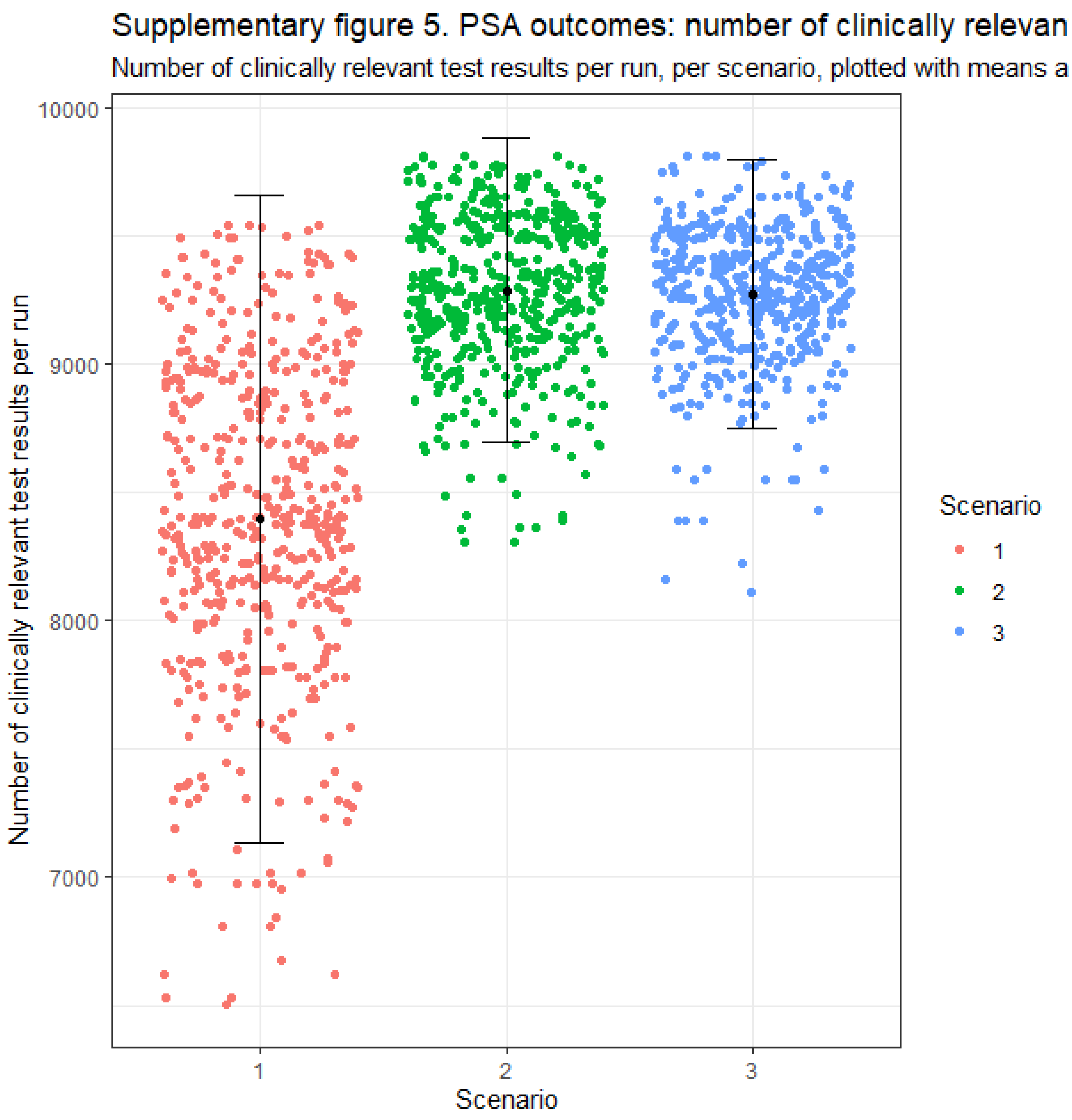

Supplementary Figure S5

Supplementary Figure S6. Cumulative mean values of cost, time and clinically relevant results, per scenario. Plots for 600 runs with 10.000 cases for determination of total number of runs in probabilistic sensitivity analysis

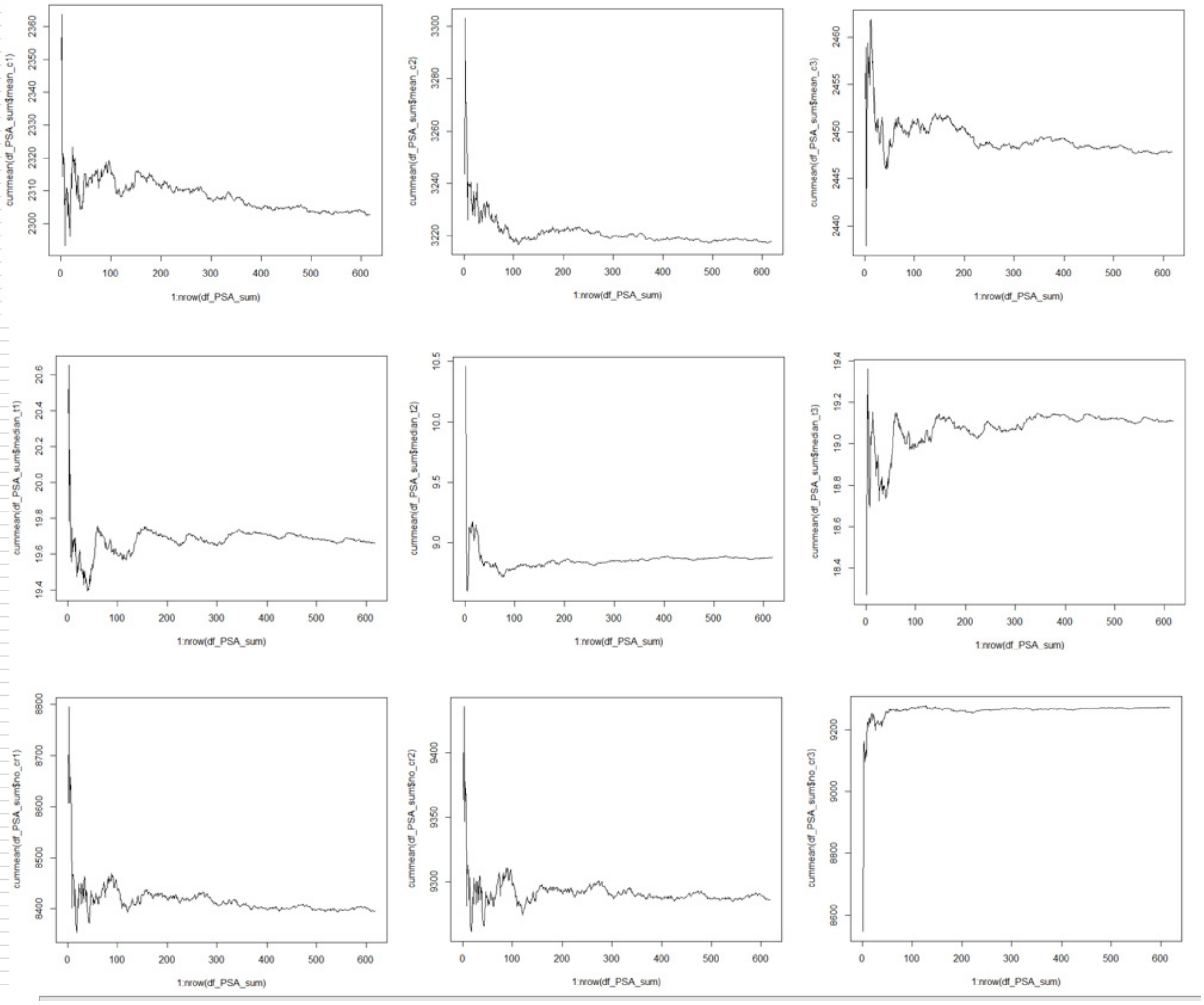

c1, cost scenario 1; c2, cost scenario 2; c3, cost scenario 3; t1, time scenario 1; t2, time scenario 2; t3, time scenario 3; cr1, clinically relevant test results scenario 1; cr2, clinically relevant test results scenario 2; c3, clinically relevant test results scenario 3.

Supplementary Figure S7. Distribution of all input parameters used in the probabilistic sensitivity analysis

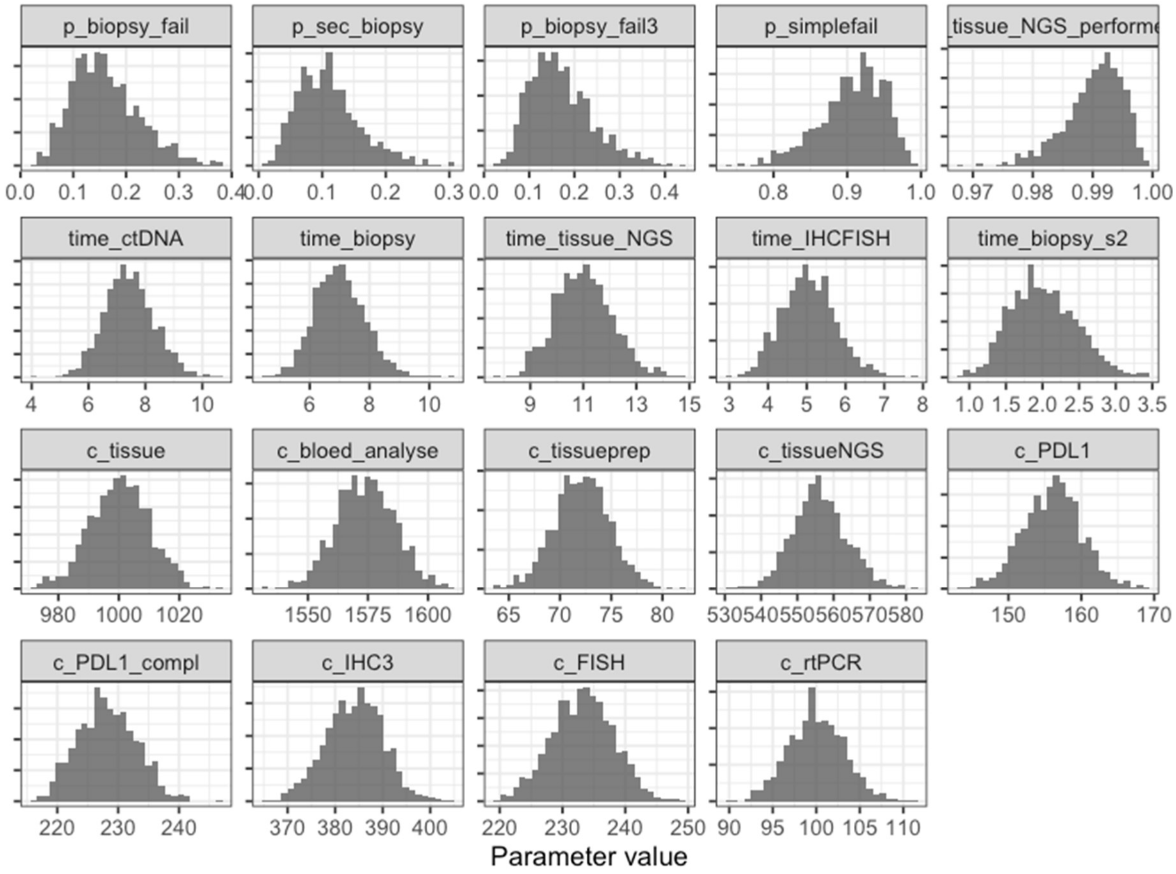

References

1. Pasmans C.T.B.; Tops B.B.J.; Steeghs E.M.P.; Coupé V.M.H.; Grünberg K.; de Jong E.K., et al. Micro-costing diagnostics in oncology: from single-gene testing to whole- genome sequencing. *Expert Rev Pharmacoecon Outcomes Res* **2021**, *21*(3):413-4.
